# A Critical Review of the Efficacy and Safety of Radiological Imaging Techniques in Pediatric Orthodontics

**DOI:** 10.31661/gmj.v13iSP1.3698

**Published:** 2024-12-31

**Authors:** Ali Rafighi, Morteza Jahanbani, Reza Mahmoudi Anzabi

**Affiliations:** ^1^ Department of Orthodontics, Faculty of Dentistry, Tabriz University of Medical Sciences, Tabriz, Iran; ^2^ Orthodontics Department, School of Dentistry, Babol University of Medical Sciences, Babol, Iran

**Keywords:** Pediatric Orthodontics, Radiographic Imaging, Cone-Beam Computed Tomography, Magnetic Resonance Imaging, Radiation Protection

## Abstract

Background: Radiological imaging plays a pivotal role in pediatric orthodontics, facilitating precise diagnosis, treatment planning, and monitoring of craniofacial anomalies and dental conditions. This narrative review critically evaluates the efficacy and safety of various imaging modalities in pediatric orthodontics, including cone-beam computed tomography (CBCT), magnetic resonance imaging (MRI), digital radiography, ultrasound, and emerging technologies such as artificial intelligence (AI) and low-intensity pulsed ultrasound (LIPUS). Materials and Methods: The review examines the literature on the use of different imaging techniques in pediatric orthodontics, focusing on their diagnostic capabilities, radiation exposure, and clinical applications. Results: While advanced imaging techniques have revolutionized orthodontic care, they present unique challenges, particularly concerning radiation exposure in pediatric patients. CBCT has emerged as an invaluable tool for complex cases, offering detailed three-dimensional visualizations crucial for assessing impacted teeth, skeletal discrepancies, and temporomandibular joint disorders. However, its higher radiation dose necessitates judicious use, guided by the ALARA (As Low As Reasonably Achievable) principle and dose optimization protocols. Alternatives such as MRI and LIPUS provide radiation-free diagnostic and therapeutic options, underscoring their growing role in pediatric orthodontics. Digital X-rays, with lower radiation doses and improved patient comfort, remain essential for routine assessments, while AI-driven technologies enhance diagnostic accuracy and streamline clinical workflows. Conclusion: The review shows the critical balance between efficacy and safety in pediatric radiological imaging. Innovations in imaging technology, such as ultra-low-dose CBCT and AI-based diagnostic tools, are paving the way for safer, more precise orthodontic care.

## Introduction

Pediatric orthodontics is at the forefront of a paradigm shift, driven by substantial advancements in diagnostic and treatment planning technologies, with radiological imaging playing a central role.

The adoption of digital tools such as intraoral scanners and 3D printing has not only revolutionized this field by facilitating highly personalized treatment plans as demonstrated by Ramdan (2023) but also significantly improved the precision of clinical outcomes [1].

In recent years, cone beam computed tomography (CBCT) has emerged as a valuable tool in orthodontic imaging, offering advantages over conventional radiographic techniques, particularly in situations where complex anatomic relationships need to be understood [2]. CBCT provides unique features and benefits, including the ability to visualize the maxillofacial skeleton in three dimensions, which is particularly useful in pediatric patients [3]. However, concerns about radiation dose and relative risk have led to the development of new techniques and protocols aimed at minimizing exposure, such as reducing the field of view (FOV) and using alternative imaging modalities like magnetic resonance imaging (MRI) [4].

Despite these advancements, the use of CBCT in pediatric orthodontics remains a topic of debate, with some studies highlighting its benefits and others raising concerns about radiation safety [5].

The development and implementation of clinical practice guidelines are critical in guiding orthodontists toward safe and effective application of these technologies for pediatric patients [6].

Additionally, case-specific studies, such as those highlighting the identification of supernumerary teeth by García et al. (2018), underscore the necessity for early and accurate diagnoses in preventing subsequent complications and optimizing therapeutic outcomes [7]. The transformative potential of three-dimensional imaging is further exemplified by its application in complex orthodontic cases [8].

This review aims to critically assess the efficacy and safety of radiological imaging techniques in advancing pediatric orthodontics. By synthesizing the latest research, it provides a comprehensive overview of their clinical applications, benefits, and inherent limitations. Through this rigorous evaluation, the paper seeks to inform evidence-based practices and stimulate further innovation in the field, ensuring optimal care outcomes for young patients.

## Radiological Imaging Modalities in Pediatric Orthodontics

Conventional X-ray

Radiological imaging in pediatric orthodontics is crucial for diagnosis, treatment planning, and monitoring. Conventional X-rays like panoramic and cephalometric images are foundational but have radiation exposure concerns. Advanced imaging, such as cone-beam computed tomography (CBCT), offers enhanced 3D visualization, improving diagnostic accuracy and treatment planning, especially for complex cases like impacted teeth and temporomandibular joint disorders [9].

Despite its advantages, low-dose spiral CT is shown to provide better image quality than traditional X-rays without significantly increasing radiation exposure [10]. Innovations in imaging technology, such as digital radiography and dose-optimized CBCT, offer promising solutions to mitigate these risks while maintaining high diagnostic quality [11][12][13]. Meanwhile, conventional X-rays continue to be indispensable in routine orthodontic practice, particularly for initial evaluations and less complex cases, due to their cost-effectiveness, availability, and lower radiation exposure. Thus, while CBCT is an invaluable tool for advanced cases, its use in pediatric orthodontics requires careful case-by-case consideration to ensure optimal patient safety and clinical outcomes.

Digital X-ray

Digital X-rays have revolutionized orthodontic diagnostics, providing precise imaging with lower radiation doses compared to conventional methods. Studies underscore their value in routine orthodontic assessments such as cephalometric and panoramic imaging, which are foundational for diagnosing malocclusions and planning treatment. Research comparing digital panoramic radiographs with CBCT has demonstrated that while digital X-rays offer lower radiation exposure, CBCT is preferred in complex cases requiring 3D visualization [14][15]; but, CBCT delivers the highest doses, particularly in adolescents, compared to panoramic and cephalographic imaging [14]. Digital imaging techniques also enable innovative diagnostic approaches, such as using thumb radiographs for assessing skeletal maturation [16].

Advancements in digital radiography have further improved patient comfort and diagnostic outcomes. For instance, phosphor plates are shown to be more patient-friendly than traditional sensors due to their flexibility, making them suitable for pediatric patients [17]. Additionally, digital radiographic records enhance data storage and sharing while reducing environmental waste, aligning with modern clinical and research needs [18]. However, practitioners are urged to adhere to the principle of keeping radiation exposure "as low as reasonably achievable" (ALARA), especially for children whose tissues are more radiosensitive [19].

Cone-Beam Computed Tomography (CBCT)

CBCT has emerged as a transformative diagnostic tool in pediatric orthodontics, offering three-dimensional (3D) imaging capabilities that surpass traditional radiography. It provides enhanced visualization of the craniofacial skeleton, teeth, and surrounding structures, making it invaluable for complex orthodontic cases, such as impacted teeth, craniofacial anomalies, and planning for orthognathic surgeries [20]. Studies demonstrate CBCT’s utility in evaluating airway morphology in obstructive sleep apnea cases and precise localization of temporary skeletal anchorage devices. However, its use should be justified based on clinical needs, considering its higher radiation dose relative to conventional X-rays [21][22].

Despite its advantages, CBCT’s higher radiation exposure has raised concerns, particularly in pediatric patients with greater radiosensitivity. Optimized imaging protocols and careful clinical judgment are essential to balance the diagnostic benefits against potential risks [23].

A study have identified specific scenarios where CBCT adds significant value, such as assessing root resorption, determining the location of unerupted teeth, and evaluating skeletal discrepancies [24]. Its indications in pediatric orthodontics are carefully categorized to ensure its application aligns with clear clinical benefits, such as pre-surgical diagnostics in cleft palate cases or advanced analysis of dentofacial anomalies [25].

Magnetic Resonance Imaging (MRI)

Magnetic Resonance Imaging (MRI) has emerged as a powerful diagnostic modality in pediatric orthodontics, providing unparalleled advantages over traditional imaging techniques, particularly for children. Unlike ionizing radiation-based tools such as X-rays or CT scans, MRI employs magnetic fields and radiofrequency waves, ensuring safety for repeated use in younger patients. MRI is particularly valuable for assessing dental and craniofacial anomalies, such as impacted teeth, supernumerary teeth, and dental abnormalities like gemination and dilacerations. Studies have demonstrated that MRI allows for precise three-dimensional (3D) localization and morphology evaluation of impacted teeth and adjacent structures without exposing children to radiation [26][27]. Its high soft-tissue contrast makes it ideal for assessing temporomandibular joint (TMJ) disorders and craniofacial abnormalities, areas where conventional radiography often lacks resolution [28].

Guidelines from professional societies like Guidelines of the Polish Orthodontic Society (PTO) and the Polish Medical Radiological Society highlight the compatibility of MRI with orthodontic materials, which typically do not interfere with imaging when made of titanium or ceramics, although stainless steel components may cause artifacts in specific areas. These artifacts can affect diagnostic accuracy, particularly in the regions close to orthodontic appliances, such as the mandible or hard palate [29][30]. Nonetheless, advances in MRI-compatible orthodontic materials and optimized protocols have significantly reduced these issues. MRI also proves useful in orthodontic treatment planning and surgical preparation, particularly for conditions requiring detailed visualization of soft tissues or complex anatomical relationships [31]. Its role in monitoring TMJ health, airway assessment, and orthodontic treatment outcomes further underscores its relevance [32].

Overall, MRI represents a highly beneficial, non-invasive tool in pediatric orthodontics, addressing key diagnostic needs with its ability to produce detailed 3D images, eliminate radiation exposure, and provide soft-tissue contrast not achievable with other modalities. Future innovations in MRI protocols and materials will likely enhance its utility, making it an indispensable adjunct in orthodontic diagnostics and treatment planning.

Ultrasound and other emerging technologies

Ultrasound has gained prominence as a non-invasive and radiation-free imaging and therapeutic modality in pediatric orthodontics. Advanced ultrasound techniques, such as low-intensity pulsed ultrasound (LIPUS), have demonstrated promising results in enhancing orthodontic treatment outcomes by accelerating bone remodeling, tooth movement, and tissue regeneration. LIPUS has been shown to reduce treatment time and complications like root resorption by stimulating osteoblast activity and promoting cementum repair [33][34]. Moreover, the application of LIPUS has been extended to mitigate orthodontically induced inflammatory root resorption, particularly in complex cases such as those involving osteoporotic conditions [35]. LIPUS has been shown to enhance bone remodeling and periodontal regeneration, helping to shorten orthodontic treatment time while reducing complications like root resorption. A study highlighted its effectiveness in both clinical and experimental settings, with LIPUS normalizing tooth movement and attenuating inflammatory root resorption in osteoporotic models, offering promising applications for postmenopausal patients [35]. Furthermore, its ability to reduce orthodontic treatment duration by nearly 50% when used with clear aligners underlines its potential as a valuable adjunct in modern orthodontics [36]. In addition to accelerating tooth movement, LIPUS significantly decreases root resorption and promotes cementum repair during orthodontic force application, further reinforcing its protective role [37].

Clinical trials and histological studies provide robust evidence supporting LIPUS’s efficacy. Low-Intensity Pulsed Ultrasound (LIPUS) enhances bone remodeling, odontoblast activity, and periodontal ligament cell counts in both normal and diabetic mandible slice organ cultures during orthodontic force application [38]. The technology is also effective in reducing pain during orthodontic treatment and enhancing patient comfort, making it particularly beneficial for long-duration therapies [39]. Its ability to prevent inflammatory root resorption and accelerate bone formation has been validated in both canine and human studies [40]. LIPUS’s applications extend beyond orthodontics, with studies demonstrating its role in periodontal regeneration, TMJ protection, and dentin-pulp repair [41]. Collectively, these findings highlight LIPUS as a versatile tool that not only accelerates orthodontic progress but also enhances the safety and quality of care in diverse clinical scenarios.

In addition to therapeutic uses, advanced imaging technologies have significantly enhanced diagnostic capabilities. Techniques such as 3D and 4D ultrasound provide high-resolution imaging for detailed anatomical assessments, particularly for soft tissue and dental anomalies. Innovations like elastography, ultrafast Doppler, and microbubble-enhanced imaging improve the evaluation of tissue stiffness and blood flow, proving beneficial for orthodontic diagnostics in children who may not tolerate lengthy or invasive procedures [42]. These technologies complement other emerging tools like focused ultrasound, which offers non-invasive therapeutic applications for managing craniofacial and musculoskeletal anomalies [43]. Together, these advancements illustrate a paradigm shift in pediatric orthodontics, integrating precision technologies to enhance diagnostic accuracy, treatment efficiency, and patient safety.

Beyond ultrasound, other emerging technologies are shaping the future of orthodontics. Artificial intelligence (AI) and machine learning are transforming diagnostics and treatment planning by analyzing dental images with greater precision, while 3D printing is enabling the creation of custom-made aligners and orthodontic appliances that enhance treatment efficiency and personalization [44]. Focused ultrasound, a non-invasive alternative to surgery, is being used to treat craniofacial anomalies and vascular conditions safely and effectively [43]. Additionally, augmented reality (AR), virtual reality (VR), and smart orthodontic devices with integrated sensors are enhancing patient engagement, improving compliance, and allowing real-time monitoring of treatment progress [45][46]. Together, these innovations are driving a paradigm shift toward precision and personalized orthodontics, ensuring safer, more effective, and patient-centered care.

## Enhancing Diagnosis, Treatment Planning, and Radiation Safety

CBCT offers unparalleled diagnostic capabilities for complex anatomical evaluations, such as identifying impacted canines, root resorption, and cleft lip/palate cases, with superior accuracy in assessing condylar morphology and spatial relationships [47]. It also enhances quantitative dental measurements like overjet, overbite, and inter-arch dimensions, making it invaluable for treatment planning [48][49]. Emerging techniques like 3D cephalometry and 3D photogrammetry are reshaping diagnostic workflows, with 3D cephalometry improving landmark identification and 3D photogrammetry providing cost-effective surface reconstructions [50][51]. Traditional lateral cephalometry remains widely used for its reliability in landmark identification and growth pattern analysis [52]. AI integration in CBCT, such as deep convolutional neural networks for automated tooth segmentation, significantly improves diagnostic efficiency and accuracy [53]. These advancements collectively enhance the precision, safety, and personalization of orthodontic care, driving better functional and esthetic outcomes. Additionally, CBCT’s integration with surgical guides enhances mini-implant placement precision, and its use in evaluating TMJ health and jaw asymmetries is critical for functional appliances and surgical interventions [47][54]. Digital radiography and 3DX dental imaging further improve diagnostic capabilities with lower radiation doses and clearer visualization [55][56][57][58]. The ALARA (As Low As Reasonably Achievable) principle is foundational, emphasizing justification, optimization, and dose minimization. Abdelkarim (2015) stresses the need for patient-specific radiographic prescriptions and adherence to ALARA to balance diagnostic benefits with radiation risks [19]. Jacobs et al. (2018) highlight that cleft palate patients face up to five times more cumulative radiation exposure, underscoring the importance of dose optimization in high-risk groups [59]. Global initiatives like Image Gently and Image Wisely, discussed by Song (2016), advocate for tailored imaging protocols and international collaboration to enhance radiation safety in pediatric populations [60]. Advances in technology, such as digital radiography and CBCT, have significantly improved dose management, offering enhanced imaging quality at reduced radiation levels. Schaetzing (2004) highlights dose-reduction techniques in modern radiography systems, including improved image processing and pediatric-specific protocols [61]. Another study emphasizes balancing CBCT’s diagnostic advantages with potential risks, especially given its higher doses compared to 2D imaging methods [62]. Aps (2013) warns that while 3D imaging offers superior diagnostic clarity, it must be used judiciously to prevent unnecessary radiation exposure, advocating for 2D imaging where clinically sufficient [63]. Efforts to minimize exposure extend beyond technology to include improved clinical practices and regulatory guidelines. Abdelkarim and Jerrold (2018) propose comprehensive risk management strategies, such as adequate radiograph interpretation and limiting imaging to clinically necessary scenarios [64]. Ludlow et al. (2008) emphasize that using digital receptors, rectangular collimation, and faster film speeds can significantly lower effective radiation doses [65]. Caird (2015) highlights the need for enhanced radiation safety awareness among clinicians, advocating for training, proper shielding, and optimized protocols to reduce exposure during diagnostic and intraoperative imaging [66]. Collectively, these strategies ensure a multifaceted approach to radiation safety in pediatric orthodontics. The integration of intraoral scanners and CAD/CAM technology has streamlined workflows, replacing traditional impressions with digital models and facilitating the fabrication of custom appliances like aligners and retainers [67][68]. Digital workflows, incorporating 3D imaging, videography, and cloud-based systems, have enhanced accessibility and collaboration. Software tools now integrate CBCT data, 3D facial imaging, and virtual treatment simulations, improving patient communication and treatment planning [69]. Digital videography captures dynamic functions such as speech and oral pharyngeal movements, enhancing diagnostic accuracy and treatment efficiency [70]. AI and machine learning (ML) have further revolutionized orthodontics. particularly in image analysis and treatment planning. AI algorithms automate cephalometric landmark identification, bone age estimation, and skeletal classification, achieving accuracy comparable to expert clinicians [71]. ML models predict treatment outcomes, such as tooth movement and alignment, offering personalized and data-driven care [72]. AI also enhances appliance design and remote monitoring, enabling real-time adjustments and precision in aligner and bracket fabrication [73]. Future trends in pediatric orthodontic imaging, including multimodal systems and advancements in genomics and proteomics, promise to provide comprehensive evaluations and personalized care [44]. Augmented reality (AR) and virtual reality (VR) enhance education, patient consultations, and surgical training [74][75][76]. Tele-orthodontics, through platforms like Dental Monitoring™, offer remote care, ensuring continuity and improving efficiency [77][78][79].

## Clinical Implications and Recommendations in Radiological Imaging for Pediatric Orthodontics

The clinical application of radiological imaging in pediatric orthodontics demands adherence to best practices, ensuring safety and efficacy. Guidelines developed by the European Academy of Paediatric Dentistry (EAPD) emphasize individualized patient-specific justification when prescribing dental radiographs. These recommendations include the ALADAIP principle (As Low As Diagnostically Achievable, being Indication-Oriented and Patient-Specific) to optimize radiation exposure and minimize risks [80][81]. Similarly, the American Academy of Oral and Maxillofacial Radiology (AAOMR) advises that CBCT should only be used when conventional methods are insufficient, emphasizing justification and dose minimization for young patients [20].

Recommendations for clinicians focus on selecting imaging techniques based on clinical necessity. Panoramic radiographs are often recommended during treatment planning, while lateral cephalograms and intraoral radiographs may be necessary for specific diagnostic tasks [82]. The British Orthodontic Society (BOS) advocates for radiographic exposure only when the benefits outweigh the risks, discouraging routine radiographs for all orthodontic cases [83]. Furthermore, advanced imaging technologies, such as 3D CBCT, are reserved for complex anatomical evaluations where conventional imaging fails to provide sufficient detail [84].

Policy implications for healthcare systems include promoting education and training for clinicians on radiation protection and imaging optimization techniques. Surveys reveal a lack of awareness among some practitioners about dose-reduction strategies, such as rectangular collimation and the Image Gently Campaign [85]. Radiologists are encouraged to lead educational initiatives to improve adherence to guidelines and promote patient safety [86]. Additionally, adopting structured decision-making tools, such as those proposed by the Children’s Oncology Group, ensures standardized imaging practices and minimizes unnecessary exposure for pediatric patients [87].

In addition to best practices, clinicians must integrate advanced technologies responsibly into their diagnostic workflows. While CBCT offers unparalleled diagnostic clarity for complex orthodontic cases, its use should be carefully justified to balance the benefits against potential risks, especially in pediatric patients. Evidence suggests that CBCT should be reserved for evaluating impacted teeth, jaw anomalies, or other conditions where conventional imaging methods are insufficient [20]. Alternatives such as panoramic radiographs and lateral cephalograms remain the mainstay for routine diagnostic needs, as they deliver lower radiation doses while providing essential information for orthodontic planning [84]. Educating clinicians about these hierarchies of imaging modalities ensures optimal patient care and safeguards against overexposure.

Healthcare systems also have a crucial role in supporting clinicians through policy development and resource allocation. The promotion of standardized imaging protocols, such as those outlined by the British Orthodontic Society (BOS), is instrumental in reducing variability in radiographic practices and ensuring adherence to the ALARA principle [83]. Further, healthcare systems should prioritize access to modern imaging tools and training programs for clinicians, promoting awareness of dose-reduction techniques like collimation and digital imaging advancements [85]. Collaborative efforts between policymakers, radiologists, and orthodontists can ensure that pediatric radiological imaging remains both effective and safe, paving the way for improved patient outcomes while mitigating long-term risks associated with radiation exposure.

## Conclusion

Radiological imaging has revolutionized pediatric orthodontics by enhancing diagnostic accuracy, treatment planning, and monitoring. Advanced techniques like CBCT, MRI, and emerging methods such as ultrasound and AI-driven analysis offer unparalleled precision, but their use must balance clinical benefits with safety risks, especially in vulnerable pediatric populations. CBCT remains essential for complex cases, requiring strict adherence to the ALARA principle to minimize radiation exposure. Digital imaging and AI technologies improve efficiency, while MRI and low-intensity pulsed ultrasound provide radiation-free alternatives. Future research should focus on refining these technologies, developing ultra-low-dose options, and conducting longitudinal studies to ensure long-term safety and efficacy. Balancing diagnostic accuracy with patient safety through innovation and evidence-based practices is crucial for optimal outcomes in pediatric orthodontics.

## Conflict of Interest

None.

## References

[R1] Ramdan KK (2023). Digital Orthodontics: An overview. MSA Dental Journal.

[R2] Scarfe WC, Azevedo B, Toghyani S, Farman AG (2017). Cone beam computed tomographic imaging in orthodontics. Australian dental journal.

[R3] Abdelkarim A (2019). Cone-beam computed tomography in orthodontics. Dentistry journal.

[R4] Tymofiyeva O, Proff PC, Rottner K, Düring M, Jakob PM, Richter EJ (2013). Diagnosis of dental abnormalities in children using 3-dimensional
magnetic resonance imaging. Journal of Oral and Maxillofacial Surgery.

[R5] Perry JL, Schleif E, Fang XM, Briley PM, McCarlie Jr (2024). Can Velopharyngeal MRI be Used in Individuals with Orthodontic
Devices.. The Cleft Palate Craniofacial Journal.

[R6] Kapetanović A, Oosterkamp BC, Lamberts AA, Schols JG (2021). Orthodontic radiology: development of a clinical practice
guideline. La radiologia medica.

[R7] Trejo-García W, Mendoza-Rodríguez M, Medina-Solís CE, Veras-Hernández MA, Lucas-Rincón SE, Casanova-Rosado JF (2018). Supernumerario invertido en paladar de un infante: reporte de un
caso clínico. Pediatría (Asunción).

[R8] Manosudprasit A, Haghi A, Allareddy V, Masoud MI (2017). Diagnosis and treatment planning of orthodontic patients with
3-dimensional dentofacial records. American Journal of Orthodontics and Dentofacial Orthopedics.

[R9] Haney E, Gansky SA, Lee JS, Johnson E, Maki K, Miller AJ, et al (2010). Comparative analysis of traditional radiographs and cone-beam
computed tomography volumetric images in the diagnosis and treatment
planning of maxillary impacted canines. American Journal of Orthodontics and Dentofacial Orthopedics.

[R10] Cordasco G, Portelli M, Militi A, Nucera R, Giudice AL, Gatto E, et al (2013). Low-dose protocol of the spiral CT in orthodontics: comparative
evaluation of entrance skin dose with traditional X-ray techniques. Progress in Orthodontics.

[R11] Yeung AW, Jacobs R, Bornstein MM (2019). Novel low-dose protocols using cone beam computed tomography in
dental medicine: a review focusing on indications, limitations, and future
possibilities. Clinical oral investigations.

[R12] Stratis A, Zhang G, Jacobs R, Bogaerts R, Bosmans H (2019). The growing concern of radiation dose in paediatric dental and
maxillofacial CBCT: an easy guide for daily practice. European Radiology.

[R13] De Felice, Di Carlo, Saccucci M, Tombolini V, Polimeni A (2019). Dental cone beam computed tomography in children: clinical
effectiveness and cancer risk due to radiation exposure. Oncology.

[R14] Lee KS, Nam OH, Kim G-T, Choi SC, Choi Y-S, Hwang E-H (2021). Radiation dosimetry analyses of radiographic imaging systems used
for orthodontic treatment: comparison among child, adolescent, and adult
patients. Oral radiology.

[R15] Goren A (2021). Dosimetry Comparison of CBCT versus Digital 2D Orthodontic
Imaging in a Pediatric Orthodontic Patient. J Dental Health Oral Res.

[R16] Moawad S (2019). Evaluation of the reliability of a practical digital dental X-ray
method for the first metacarpophalangeal joint to assist the skeletal age in
peripubertal orthodontic period. International Orthodontics.

[R17] Russo JM, Russo JA, Guelmann M (2006). Digital radiography: a survey of pediatric dentists. Journal of dentistry for children.

[R18] Alqahtani J, Alhemaid G, Alqahtani H, Abughandar A, AlSaadi R, Algarni I, et al (2022). Digital Diagnostics and Orthodontic Practice. J Healthc Sci.

[R19] Abdelkarim AA (2015). Appropriate use of ionizing radiation in orthodontic practice and
research. American Journal of Orthodontics and Dentofacial Orthopedics.

[R20] Scarfe W, Azevedo B, Toghyani S, Farman A (2017). Cone beam computed tomographic imaging in orthodontics. Australian dental journal.

[R21] Lenza M, Lenza MdO, Dalstra M, Melsen B, Cattaneo P (2010). An analysis of different approaches to the assessment of upper
airway morphology: a CBCT study. Orthodontics & craniofacial research.

[R22] Gurgel ML, Junior CC, Cevidanes LHS, de Barros, Carvalho FSR, Kurita LM, et al (2023). Methodological parameters for upper airway assessment by
cone-beam computed tomography in adults with obstructive sleep apnea: a
systematic review of the literature and meta-analysis. Sleep and Breathing.

[R23] Horner K, O'Malley L, Taylor K, Glenny A-M (2015). Guidelines for clinical use of CBCT: a review. Dentomaxillofacial radiology.

[R24] Hodges RJ, Atchison KA, White SC (2013). Impact of cone-beam computed tomography on orthodontic diagnosis
and treatment planning. American journal of orthodontics and dentofacial orthopedics.

[R25] Theys S, Olszewski R (2022). Cone beam computed tomography (CBCT) in pediatric dentistry. Nemesis Negative Effects in Medical Sciences Oral and Maxillofacial
Surgery.

[R26] Tymofiyeva O, Rottner K, Jakob P, Richter E-J, Proff P (2010). Three-dimensional localization of impacted teeth using magnetic
resonance imaging. Clinical oral investigations.

[R27] Tymofiyeva O, Proff PC, Rottner K, Düring M, Jakob PM, Richter E-J (2013). Diagnosis of dental abnormalities in children using 3-dimensional
magnetic resonance imaging. Journal of Oral and Maxillofacial Surgery.

[R28] Patel A, Bhavra G, O'Neill J (2006). MRI scanning and orthodontics. Journal of Orthodontics.

[R29] Dobai A, Dembrovszky F, Vízkelety T, Barsi P, Juhász F, Dobó-Nagy C (2022). MRI compatibility of orthodontic brackets and wires: systematic
review article. BMC Oral Health.

[R30] Hasanin M, Kaplan SE, Hohlen B, Lai C, Nagshabandi R, Zhu X, et al (2019). Effects of orthodontic appliances on the diagnostic capability of
magnetic resonance imaging in the head and neck region: A systematic review. International Orthodontics.

[R31] Aizenbud D, Hazan-Molina H, Einy S, Goldsher D (2012). Craniofacial Magnetic Resonance Imaging With a Gold Solder–Filled
Chain-Like Wire Fixed Orthodontic Retainer. Journal of Craniofacial Surgery.

[R32] Shivam R, Rogers S, Drage N (2021). An Evidence-based Protocol for the Management of Orthodontic
Patients Undergoing MRI Scans. Orthodontic Update.

[R33] Mansjur KQ, Nasir M, Paramma ZI, Wahyuni I (2021). Low intensity pulsed ultrasound in orthodontic tooth movement. Makassar Dental Journal.

[R34] Liu Z, Xu J, E L, Wang D (2012). Ultrasound enhances the healing of orthodontically induced root
resorption in rats. The Angle Orthodontist.

[R35] Dahhas FY, El-Bialy T, Afify AR, Hassan AH (2016). Effects of low-intensity pulsed ultrasound on orthodontic tooth
movement and orthodontically induced inflammatory root resorption in
ovariectomized osteoporotic rats. Ultrasound in medicine & biology.

[R36] Kaur H, El-Bialy T (2020). Shortening of overall orthodontic treatment duration with
low-intensity pulsed ultrasound (LIPUS). Journal of Clinical Medicine.

[R37] Raza H, Major P, Dederich D, El-Bialy T (2016). Effect of low-intensity pulsed ultrasound on orthodontically
induced root resorption caused by torque: a prospective, double-blind,
controlled clinical trial. The Angle Orthodontist.

[R38] Alshihah N, Alhadlaq A, El-Bialy T, Aldahmash A, Bello IO (2020). The effect of low intensity pulsed ultrasound on dentoalveolar
structures during orthodontic force application in diabetic ex-vivo model. Archives of Oral Biology.

[R39] Badiee M, Tehranchi A, Behnia P, Khatibzadeh K (2021). Efficacy of low-intensity pulsed ultrasound for orthodontic pain control: a randomized clinical trial. Frontiers in Dentistry.

[R40] Al-Daghreer S, Doschak M, Sloan AJ, Major PW, Heo G, Scurtescu C, et al (2014). Effect of low-intensity pulsed ultrasound on orthodontically
induced root resorption in beagle dogs. Ultrasound in medicine & biology.

[R41] Wei Y, Guo Y (2022). Clinical applications of low-intensity pulsed ultrasound and its
underlying mechanisms in dentistry. Applied Sciences.

[R42] Hwang M, Piskunowicz M, Darge K (2019). Advanced ultrasound techniques for pediatric imaging. Pediatrics.

[R43] Janwadkar R, Leblang S, Ghanouni P, Brenner J, Ragheb J, Hennekens CH, et al (2022). Focused ultrasound for pediatric diseases. Pediatrics.

[R44] Jheon A, Oberoi S, Solem R, Kapila S (2017). Moving towards precision orthodontics: An evolving paradigm shift
in the planning and delivery of customized orthodontic therapy. Orthodontics & craniofacial research.

[R45] Huang T-K, Yang C-H, Hsieh Y-H, Wang J-C, Hung C-C (2018). Augmented reality (AR) and virtual reality (VR) applied in
dentistry. The Kaohsiung journal of medical sciences.

[R46] Al-Ansi AM, Jaboob M, Garad A, Al-Ansi A (2023). Analyzing augmented reality (AR) and virtual reality (VR) recent
development in education. Social Sciences & Humanities Open.

[R47] De Grauwe, Ayaz I, Shujaat S, Dimitrov S, Gbadegbegnon L, Vande Vannet, et al (2019). CBCT in orthodontics: a systematic review on justification of
CBCT in a paediatric population prior to orthodontic treatment. European journal of orthodontics.

[R48] Baumgaertel S, Palomo JM, Palomo L, Hans MG (2009). Reliability and accuracy of cone-beam computed tomography dental
measurements. American journal of orthodontics and dentofacial orthopedics.

[R49] Sang Y-H, Hu H-C, Lu S-H, Wu Y-W, Li W-R, Tang Z-H (2016). Accuracy assessment of three-dimensional surface reconstructions
of in vivo teeth from cone-beam computed tomography. Chinese medical journal.

[R50] Pittayapat P, Limchaichana‐Bolstad N, Willems G, Jacobs R (2014). Three‐dimensional cephalometric analysis in orthodontics: a
systematic review. Orthodontics & craniofacial research.

[R51] Pojda D, Tomaka AA, Luchowski L, Tarnawski M (2021). Integration and application of multimodal measurement techniques:
relevance of photogrammetry to orthodontics. Sensors.

[R52] Heil A, Lazo Gonzalez, Hilgenfeld T, Kickingereder P, Bendszus M, Heiland S, et al (2017). Lateral cephalometric analysis for treatment planning in
orthodontics based on MRI compared with radiographs: A feasibility study in
children and adolescents. PloS one.

[R53] Ayidh Alqahtani, Jacobs R, Smolders A, Van Gerven, Willems H, Shujaat S, et al (2023). Deep convolutional neural network-based automated segmentation
and classification of teeth with orthodontic brackets on cone-beam
computed-tomographic images: a validation study. European Journal of Orthodontics.

[R54] Kim S-H, Choi Y-S, Hwang E-H, Chung K-R, Kook Y-A, Nelson G (2007). Surgical positioning of orthodontic mini-implants with guides
fabricated on models replicated with cone-beam computed tomography. American Journal of orthodontics and dentofacial orthopedics.

[R55] Horner K, Barry S, Dave M, Dixon C, Littlewood A, Pang CL, et al (2020). Diagnostic efficacy of cone beam computed tomography in
paediatric dentistry: a systematic review. European Archives of Paediatric Dentistry.

[R56] Durão AR, Pittayapat P, Rockenbach MIB, Olszewski R, Ng S, Ferreira AP, et al (2013). Validity of 2D lateral cephalometry in orthodontics: a systematic
review. Progress in orthodontics.

[R57] McClure SR, Sadowsky PL, Ferreira A, Jacobson A (2005). Reliability of digital versus conventional cephalometric radiology: a comparative evaluation of landmark identification error. Seminars in Orthodontics.

[R58] Nakajima A, Sameshima GT, Arai Y, Homme Y, Shimizu N, Dougherty Sr (2005). Two-and three-dimensional orthodontic imaging using limited cone
beam–computed tomography. The Angle Orthodontist.

[R59] Jacobs R, Pauwels R, Scarfe WC, De Cock, Dula K, Willems G, et al (2018). Pediatric cleft palate patients show a 3-to 5-fold increase in
cumulative radiation exposure from dental radiology compared with an age-and
gender-matched population: a retrospective cohort study. Clinical oral investigations.

[R60] Song H (2016). Current global and Korean issues in radiation safety of nuclear
medicine procedures. Annals of the ICRP.

[R61] Schaetzing R (2004). Management of pediatric radiation dose using Agfa computed
radiography. Pediatric radiology.

[R62] Pawlowski JM, Ding GX (2014). An algorithm for kilovoltage x-ray dose calculations with
applications in kV-CBCT scans and 2D planar projected radiographs. Physics in Medicine & Biology.

[R63] Aps J (2013). Three-dimensional imaging in paediatric dentistry: a must-have or
you’re not up-to-date. European Archives of Paediatric Dentistry.

[R64] Abdelkarim A, Jerrold L (2018). Clinical considerations and potential liability associated with
the use of ionizing radiation in orthodontics. American journal of orthodontics and dentofacial orthopedics.

[R65] Ludlow JB, Davies-Ludlow LE, White SC (2008). Patient risk related to common dental radiographic examinations:
the impact of 2007 International Commission on Radiological Protection
recommendations regarding dose calculation. The journal of the American Dental association.

[R66] Caird MS (2015). Radiation safety in pediatric orthopaedics. Journal of Pediatric Orthopaedics.

[R67] Quintero JC, Trosien A, Hatcher D, Kapila S (1999). Craniofacial imaging in orthodontics: historical perspective,
current status, and future developments. The Angle Orthodontist.

[R68] Francisco I, Ribeiro MP, Marques F, Travassos R, Nunes C, Pereira F, et al (2022). Application of three-dimensional digital technology in
orthodontics: the state of the art. Biomimetics.

[R69] Shelke DD, Jadhav VV, Jaiswal A, Gandhi V (2022). Review on digital orthodontics. J Pharmaceut Res Int.

[R70] Nc SC, Kalidass DSP, Davis D, Kishore S, Suvetha S (2021). Orthodontics in the Era of Digital Innovation--A Review. Journal of Evolution of Medical and Dental Sciences.

[R71] Mohammad-Rahimi H, Nadimi M, Rohban MH, Shamsoddin E, Lee VY, Motamedian SR (2021). Machine learning and orthodontics, current trends and the future opportunities: A scoping review. American Journal of Orthodontics and Dentofacial Orthopedics.

[R72] Albalawi F, Alamoud KA (2022). Trends and application of artificial intelligence technology in
orthodontic diagnosis and treatment planning—A review. Applied Sciences.

[R73] Kapila S, Vora SR, Rengasamy Venugopalan, Elnagar MH, Akyalcin S (2023). Connecting the dots towards precision orthodontics. Orthodontics & craniofacial research.

[R74] Rao GKL, Iskandar YHP, Mokhtar N (202).

[R75] Joda T, Gallucci G, Wismeijer D, Zitzmann NU (2019). Augmented and virtual reality in dental medicine: A systematic
review. Computers in biology and medicine.

[R76] Ayoub A, Pulijala Y (2019). The application of virtual reality and augmented reality in Oral
& Maxillofacial Surgery. BMC Oral Health.

[R77] Hansa I, Semaan SJ, Vaid NR, Ferguson DJ (2018). Remote monitoring and “Tele-orthodontics”: Concept, scope and applications. Seminars in Orthodontics.

[R78] Saccomanno S, Quinzi V, Sarhan S, Laganà D, Marzo G (2020). Perspectives of tele-orthodontics in the COVID-19 emergency and
as a future tool in daily practice. European journal of paediatric dentistry.

[R79] Wafaie K, Rizk MZ, Basyouni ME, Daniel B, Mohammed H (2023). Tele-orthodontics and sensor-based technologies: a systematic
review of interventions that monitor and improve compliance of orthodontic
patients. European journal of orthodontics.

[R80] Masood H, Rossouw PE, Barmak AB, Malik S (2023). Tele-orthodontics education model for orthodontic residents: A
preliminary study. Journal of Telemedicine and Telecare.

[R81] Kühnisch J, Anttonen V, Duggal M, Spyridonos ML, Rajasekharan S, Sobczak M, et al (2020). Best clinical practice guidance for prescribing dental
radiographs in children and adolescents: an EAPD policy document. European Archives of Paediatric Dentistry.

[R82] Stervik C, Lith A, Westerlund A, Ekestubbe A (2021). Choice of radiography in orthodontic treatment on children and
adolescents: A questionnaire‐based study performed in Sweden. European Journal of Oral Sciences.

[R83] Turpin DL (2008). British Orthodontic Society revises guidelines for clinical
radiography. American journal of orthodontics and dentofacial orthopedics.

[R84] Silva MAG, Wolf U, Heinicke F, Bumann A, Visser H, Hirsch E (2008). Cone-beam computed tomography for routine orthodontic treatment planning: a radiation dose evaluation. American Journal of Orthodontics and Dentofacial Orthopedics.

[R85] Campbell RE, Wilson S, Zhang Y, Scarfe WC (2020). A survey on radiation exposure reduction methods including
rectangular collimation for intraoral radiography by pediatric dentists in
the United States. The Journal of the American Dental Association.

[R86] Pfeifer CM (2020). Promoting imaging appropriateness in pediatric radiology. Pediatric radiology.

[R87] Hua Ch, Vern‐Gross TZ, Hess CB, Olch AJ, Alaei P, Sathiaseelan V, et al (2020). Practice patterns and recommendations for pediatric image‐guided
radiotherapy: a Children's Oncology Group report. Pediatric blood & cancer.

